# Low body mass is associated with reduced left ventricular mass in Chinese elderly with severe COPD

**DOI:** 10.1038/s41598-021-92212-0

**Published:** 2021-06-22

**Authors:** Jing Zhang, Gang Li, Jari A. Laukkanen, Cheng Liu, Xing Song, Yuqi Zhu

**Affiliations:** 1 Division of Cardiology, Department of Geriatrics, The First Affiliated Hospital of Chongqing Medical University, No. 1 Youyi Road, Yuzhong District, Chongqing, 400016 China; 2 Institute of Public Health and Clinical Nutrition, University of Eastern Finland, Kuopio, Finland; 3 Department of Medicine, Institute of Clinical Medicine, University of Eastern Finland, Kuopio, Finland; 4 Department of Medicine, Central Finland Health Care District, Jyvaskyla, Finland

**Keywords:** Cardiovascular diseases, Respiratory tract diseases, Cardiology, Diseases, Medical research

## Abstract

There is limited information on the association of body mass index (BMI) with left ventricular (LV) remodeling corresponding to severity of reduced lung function in patients with chronic obstructive pulmonary disease (COPD). Therefore, we investigated whether BMI is associated with cardiac atrial and ventricular dimensions according to severity of lung functional impairment in Chinese COPD elderly. A total of 563 hospitalized COPD patients with lung function impairment and 184 patients with non-COPD (aged 65–92 years) were collected retrospectively in a cross-sectional study in a university affiliated tertiary hospital in China. BMI and cardiac echocardiographic parameters were compared according to severity of lung functional impairment in COPD patients. BMI was 22.9 ± 3.9 kg/m^2^ in COPD patients, 24.0 ± 4.1 kg/m^2^ in non-COPD patients respectively. Reduced BMI, LV mass index, LV wall thickness and left atrial diameter, and dilated right ventricle (RV) existed in COPD patients with severe lung dysfunction as compared the COPD patients with mild to moderate lung functional reduction and non-COPD patients (*P* < 0.05), while there were no differences in BMI and echocardiographic parameters between the COPD patients with mild to moderate lung functional decline and non-COPD patients (*P* > 0.05). Logistic regression analysis showed that low BMI (BMI < 18.5 kg/m^2^) was correlated with reduced LV mass and wall thickness, dilated RV and reduced lung function in the COPD patients with severe lung dysfunction. In conclusion, this study demonstrates that lower BMI is associated not only with dilated RV and impaired pulmonary function, but also it is related to reduced LV mass in Asian COPD elderly with severe lung dysfunction.

Chronic obstructive pulmonary disease (COPD), as a prevalent progressive disease, is a leading cause of all-cause death in the adult population. Previous investigations found that the prevalence of COPD is higher among elderly subjects and women as compared with younger populations and men respectively^1–3^. It has been reported that low body mass index (BMI), bodyweight loss and cachexia are correlated with impaired lung function, increased all-cause mortality in COPD patients^4–8^. In addition, studies have confirmed that lower BMI may be related to increased cardiovascular events and cardiac mortality^9,10^. Although it is well known that long-term COPD will be complicated more often with lung functional impairment and pulmonary heart disease, which is characteristically manifested as right-sided atrial and ventricular enlargement, several studies have reported that reduced left-sided cardiac filling (preload) was found in COPD patients^11,12^, and the association of impaired lung function with left-sided cardiac atrial and ventricular dimensional changes was more often seen in hypertensive women^13^. However, up to now, it is unclear whether BMI is related to the left-sided cardiac structural remodeling corresponding to different severity of lung functional reduction in COPD patients. Therefore, in this study, we analysed data from medical records to investigate if BMI is associated with cardiac remodeling according to different severity of lung functional impairment in Chinese COPD elderly.

## Methods

### Patients

Data were collected retrospectively in a cross-sectional observatory study from the department of respiratory diseases in the First Affiliated Hospital of Chongqing Medical University, Chongqing, China. After referring to the exclusion criteria listed below, a total of 747 elderly aged 65 to 92 years (500 males, 247 females) were suspected with COPD and hospitalized due to their complaints of chronic cough and dyspnea with phlegm for more than two years. Then, after patient’s condition became relieved, they underwent a post-bronchodilator spirometry test from January 2014 to 2018 December. Among them, 184 subjects were diagnosed with non-COPD (forced expiratory volume in one second (FEV1)/forced vital capacity (FVC) ≥ 70%), 563 with COPD (FEV1/FVC < 70%), including 77 with mild lung functional reduction (FEV1% pred. ≥ 80%), 206 with moderate lung functional reduction (80% > FEV1% pred. ≥ 50%), and 280 with severe lung functional reduction (FEV1% pred. < 50%) according to the GOLD COPD criteria, which is commonly used nowadays^14^. Acute exacerbation of COPD was defined as a self-reported symptomatic exacerbation treated with antibiotics and/or intravenous corticosteroids, and requiring emergency departmental visitation or hospital admission.

Some common comorbidities with COPD were included in this study. The criteria used for the diagnosis of primary hypertension, type 2 diabetes mellitus (DM), gout, coronary heart disease and stroke were the same as described previously^15–18^. Emphysema was confirmed by computed tomography or x-ray examination^12^. The study was carried out in accordance with the declaration of Helsinki for research involving humans. This study received approval from the ethics committee of the First Affiliated Hospital of Chongqing Medical University (No. 2020-327), while the ethics committee waived the need for patients’ written informed consent because of the anonymous nature of the clinical data acquired retrospectively.

We excluded patients with pulmonary interstitial disease, pulmonary embolism, pulmonary abscess and tuberculosis, bronchiectasis, restrictive lung disease, lung cancer, pulmonary lobectomy, sleep apnea hypopnea syndrome, cardiac valvular disease, dilated, hypertrophic or restricted primary cardiomyopathy, congenital heart disease, atrial fibrillation, persistent rapid arrhythmias and malignant tumor.

### Assessment of body mass index

BMI = body weight, kg/(height, m)^2^. Overweight obesity, normal weight and underweight were categorized according to World Health Organization criteria for Asian populations^19^. Overweight and obesity refers BMI ≥ 23 kg/m^2^; Normal weight: 23 kg/m^2^ > BMI ≥ 18.5 kg/m^2^; Underweight: BMI < 18.5 kg/m^2^^19^.

### Measurement of cardiac structure and function by echocardiography

According to American Society of Echocardiography leading-edge method, transthoracic color doppler echocardiographic studies were performed and read by experienced cardiologists in a single core laboratory in our hospital, with a Vivid echocardiographic system (General Electric Co., Fairfield, CT, USA), which was equipped with a 3.5-MHz sector scan transducer^20^. Two-dimensional guided M-mode measurement of right ventricular (RV) diameter, interventricular septal thickness, left ventricular (LV) posterior wall thickness and end-diastolic inner diameter were examined at end-diastole from the parasternal long-axis view; Right atrial (RA), left atrial (LA) and LV end-systolic inner diameter were measured at end-systole from the parasternal long-axis view; The methods for the calculation of LV mass index and ejection fraction were the same as described previously^16,17,20^.

### Measurement of blood glucose, lipids and eGFR

The methods for the measurement of plasma glucose, total cholesterol, low density lipoprotein cholesterol, high density lipoprotein cholesterol (HDL-C), triglyceride, and the calculation of eGFR (estimated glomerular filtration rate) were the same as described previously^16,18,21^.

### Statistical analysis

Categorical data were summarized as percentages and compared using Fisher exact or Chi-square tests. Continuous variables were expressed as mean values ± standard deviation (SD). Depending on the variable normal distribution or not, the statistical differences were evaluated by one-way ANOVA (analysis of variance) or Kruskal–Wallis H test for multiple comparisons, followed by post hoc analysis using the LSD (least significance difference) or Mann–Whitney U test between the groups. Pearson or Spearman univariate correlation and dichotomized variate logistic regression were utilized for data analysis. Statistical analysis was performed by SPSS (statistical package for social science) 22.0 software package (IBM Company, Chicago, Illinois 60606, USA). A 2-tailed value of *P* < 0.05 was considered to be statistically significant. Given the high collinearity between emphysema and the spirometric parameter (residual volume (RV)/total lung capacity (TLC)), only RV/TLC was included in the regression analysis.

## Results

### Comparison of BMI, cardiac structure and clinical characteristics among different lung dysfunction categories in COPD

The mean age and BMI (SD) were 74.6 ± 6.6 years and 22.9 ± 3.9 kg/m^2^ in COPD patients, 73.0 ± 7.4 years and 24.0 ± 4.1 kg/m^2^ in non-COPD patients; respectively (Both *P* < 0.05, Table 1).

**Table 1:** Comparison of clinical characteristics among different lung dysfunction categories in COPD.

Variable	Non-COPD (n = 184)	Lung functional impairment in COPD			*P* value
		Mild (n = 77)	Moderate (n = 206)	Severe (n = 280)	
Age, year	73.0 ± 7.4	76.5 ± 6.1*	75.1 ± 6.9*	73.6 ± 6.4 ^†‡^	< 0.001
Sex, M/F, n	87/97	40/37	140/66*^†^	233/47*^†‡^	< 0.001
BMI, kg/m^2^	24.0 ± 4.1	23.8 ± 3.7	23.6 ± 4.0	22.1 ± 3.8*^†‡^	< 0.001
Underweight, n (%)	16 (8.7)	6 (7.8)	20 (9.7)	51 (18.2)*^†‡^	0.003
Smoking, n (%)	55 (30.1)	31 (40.3)	122 (59.8)*^†^	210 (75.0)*^†‡^	< 0.001
Drinking, n (%)	50 (27.2)	21 (27.3)	73 (35.8)	117 (41.8)*^†^	0.030
Disease duration, year	9.0 ± 10.2	10.3 ± 12.8	10.5 ± 11.8	14.6 ± 11.4*^†‡^	< 0.001
Exacerbator, n (%)	79 (42.9)	55 (71.4)*	158 (76.7)*	245 (87.5)*^†‡^	< 0.001
Emphysema, n (%)	36 (19.6)	29 (37.7)*	112 (54.4)*^†^	200 (71.4)*^†‡^	< 0.001
FEV1, % pred	99.9 ± 21.3	93.4 ± 12.0*	62.0 ± 8.2*^†^	34.4 ± 8.6*^†‡^	< 0.001
FEV1/FVC, %	78.1 ± 6.2	62.4 ± 5.3*	53.3 ± 8.6*^†^	39.4 ± 8.8*^†‡^	< 0.001
RV/TLC, %	45.4 ± 8.1	47.5 ± 7.3	53.0 ± 8.9*^†^	62.5 ± 8.2*^†‡^	< 0.001
DLCO, % pred	70.4 ± 18.8	63.2 ± 17.2*	52.8 ± 20.8*^†^	31.1 ± 15.9*^†‡^	< 0.001
RV diameter, mm	19.1 ± 2.0	19.1 ± 2.4	19.3 ± 2.1	20.0 ± 3.1*^†‡^	0.001
RA diameter, mm	33.8 ± 4.8	33.9 ± 5.1	34.2 ± 4.3	34.5 ± 5.2	0.501
LA diameter, mm	30.4 ± 5.0	30.2 ± 4.8	29.7 ± 4.7	28.9 ± 3.8*^†^	0.003
LV ESD, mm	31.2 ± 4.5	31.1 ± 3.6	30.9 ± 4.1	30.4 ± 3.5	0.171
LV EDD, mm	47.7 ± 4.7	47.2 ± 4.9	47.0 ± 4.9	46.5 ± 4.4	0.173
IV septum, mm	10.7 ± 1.4	10.5 ± 1.0	10.5 ± 1.2	10.3 ± 1.2*	0.004
LV PWT, mm	10.5 ± 1.3	10.3 ± 1.1	10.3 ± 1.0	10.2 ± 1.1*	0.032
LV mass index, g/m^2.7^	53.4 ± 14.4	53.2 ± 11.8	49.4 ± 12.5	46.4 ± 11.4*^†‡^	< 0.001
LV ejection fraction, %	62.8 ± 5.4	63.4 ± 4.9	63.8 ± 4.9	63.7 ± 3.9	0.187
E/A < 1, n (%)	117 (67.5)	52 (63.6)	126 (63.9)	179 (61.2)	0.285
HT, n (%)	111 (60.3)	46 (59.7)	110 (53.4)	124 (44.3)*^†‡^	0.003
SBP, mmHg	138.6 ± 20.2	137.8 ± 21.6	137.3 ± 19.8	136.0 ± 19.3	0.783
DBP, mmHg	78.8 ± 11.5	77.6 ± 12.0	75.8 ± 14.7	75.3 ± 12.3*	0.017
Type 2 DM, n (%)	51 (27.7)	14 (18.2)	39 (18.9)*	44 (15.7)*	0.015
FBG, mmol/L	5.9 ± 1.9	5.9 ± 1.8	5.9 ± 1.7	5.9 ± 1.9	0.998
TC, mmol/L	4.2 ± 1.0	4.1 ± 1.1	4.0 ± 0.9	4.0 ± 0.9	0.335
LDL-C, mmol/L	2.6 ± 0.8	2.5 ± 0.9	2.4 ± 0.8	2.4 ± 0.7	0.158
HDL-C, mmol/L	1.2 ± 0.4	1.3 ± 0.4	1.3 ± 0.4	1.4 ± 0.5	0.152
TG, mmol/L	1.5 ± 1.1	1.3 ± 0.7	1.1 ± 0.6*	1.0 ± 0.5*^†^	< 0.001
Gout, n (%)	7 (3.8)	2 (2.6)	5 (2.4)	2 (0.7)	0.087
eGFR, ml/min/1.73m^2^	72.3 ± 20.4	77.6 ± 17.0*	77.7 ± 17.9*	81.9 ± 16.2*^†‡^	< 0.001
CHD, n (%)	58 (31.5)	24 (31.2)	57 (27.7)	55 (19.6)*^†‡^	0.017
Stroke, n (%)	46 (25.0)	10 (13.0)	43 (20.9)	36 (12.9)**	0.032
LABA, n (%)	9 (4.9)	22 (28.6)*	113 (54.9)*^†^	220 (78.6)*^†‡^	< 0.001
LAMA, n (%)	3 (1.6)	20 (26.0)*	86 (41.7)*^†^	167 (59.6)*^†‡^	< 0.001

Values presented as mean ± SD or n (%).* *p* < 0.05 versus non-COPD group; † *p* < 0.05 versus mild lung functional impairment group; ‡ *p* < 0.05 versus moderate lung functional impairment group. COPD, chronic obstructive pulmonary disease; BMI, body mass index; FEV1, forced expiratory volume in one second; FVC, forced vital capacity; RV, residual volume; TLC, total lung capacity; DLCO, diffusing capacity of carbon monoxide; RV, right ventricular; RA, right atrial; LA, left atrial; LV ESD, left ventricular end-systolic diameter; LV EDD, left ventricular end-diastolic diameter; IV, interventricular thickness at the end-diastole; LV PWT, left ventricular posterior wall thickness at the end-diastole; LV, left ventricular; E/A: transmitral peak early (E) and late (A) filling velocity ratio; HT, hypertension; SBP, systolic blood pressure; DBP, diastolic blood pressure; DM, diabetes mellitus; FPG, fast plasma glucose; TC, total cholesterol; LDL-C, low density lipoprotein cholesterol; HDL-C, high density lipoprotein cholesterol; TG, triglyceride; eGFR, estimated glomerular filtration rate; CHD, coronary heart disease; LABA, Long-acting β2 adrenergic receptor agonist; LAMA, Long-acting muscarinic antagonist.

BMI, LA diameter, LV mass index and wall thickness were lower, and RV diameter was higher in COPD patients with severe lung functional reduction as compared with COPD patients with mild to moderate lung functional reduction and non-COPD patients (*P* < 0.05, Table 1). There was no significant difference in BMI, cardiac structure and function between COPD patients with mild to moderate lung functional reduction and non-COPD patients (*P* > 0.05, Table 1).

The male sex, age, eGFR, prevalence of cigarette smoking and alcohol drinking were higher in COPD patients with mild, moderate or severe lung functional reduction as compared with non-COPD patients (All *P* < 0.05, Table 1); Plasma triglyceride, diastolic blood pressure, prevalence of hypertension, type 2 DM, coronary heart disease and stroke were lower in COPD patients with mild, moderate or severe lung functional reduction as compared with non-COPD patients (All *P* < 0.05, Table 1).

### Echocardiographic and clinical parameters among different BMI categories in patients with severe COPD

Comparison of echocardiographic, lung function and clinical parameters among different BMI categorized groups was performed in COPD patients with severe lung functional reduction (n = 280), as considering that the changes in echocardiographic parameters and reduced BMI existed in COPD patients with severe lung functional reduction. LA diameter, LV mass index and wall thickness, lung function, plasma total cholesterol, systolic blood pressure, prevalence of hypertension and type 2 DM were lower in underweight patients as compared with normal weight or overweight obesity patients in COPD with severe lung functional reduction (All *P* < 0.05, Table 2); While the RV and RA diameter, HDL-C and eGFR were higher in underweight patients as compared with normal weight or overweight obesity patients in COPD with severe lung functional reduction (All *P* < 0.05, Table 2).

**Table 2:** Comparison of clinical parameters among different BMI categories and correlations in patients with severe COPD (n = 280).

Variable	Overweight obesity (n = 114)	Normal weight (n = 115)	Under weight (n = 51)	*r*	*P* value
Age, year	73.2 ± 6.2	74.6 ± 6.6	72.5 ± 6.5	0.055	0.357
Sex, M/F, n	93/21	95/20	45/6	− 0.089	0.139
BMI, kg/m^2^	25.7 ± 2.4	20.9 ± 1.3*	16.7 ± 1.5*^†^	1.000	< 0.001
Smoking, n (%)	82 (71.9)	86 (74.8)	42 (82.4)	− 0.071	0.233
Drinking, n (%)	53 (46.5)	45 (39.1)	19 (37.3)	0.070	0.241
COPD duration, year	14.1 ± 11.0	15.3 ± 12.5	13.8 ± 9.5	0.037	0.543
Exacerbator, n (%)	100 (87.7)	100 (87.0)	45 (88.2)	− 0.032	0.590
Emphysema, n (%)	73 (64.0)	82 (71.3)	45 (88.2)*^†^	− 0.232	< 0.001
FEV1, % pred	36.0 ± 8.2	34.1 ± 8.9	31.6 ± 8.5*^†^	0.198	0.001
FEV1/FVC, %	41.7 ± 9.2	39.0 ± 8.2*	35.2 ± 7.4*^†^	0.337	< 0.001
RV/TLC, %	61.2 ± 7.5	62.7 ± 8.3	65.5 ± 9.2*	− 0.167	0.018
DLCO, % pred	33.4 ± 14.6	31.2 ± 16.4	24.3 ± 17.0*^†^	0.216	0.002
RV diameter, mm	19.4 ± 2.2	20.0 ± 3.4	20.3 ± 3.2*	− 0.132	0.027
RA diameter, mm	32.6 ± 4.2	34.5 ± 5.6	35.0 ± 4.9*	− 0.176	0.003
LA diameter, mm	30.1 ± 3.7	28.4 ± 3.6*	27.2 ± 3.5*	0.360	< 0.001
LV ESD, mm	30.8 ± 2.9	30.3 ± 3.3	29.8 ± 5.0	0.117	0.052
LV EDD, mm	47.1 ± 3.9	46.4 ± 4.3	45.4 ± 5.6	0.103	0.060
IV septum, mm	10.6 ± 1.0	10.3 ± 1.1*	9.5 ± 1.1*^†^	0.349	< 0.001
LV PWT, mm	10.6 ± 1.0	10.2 ± 1.1*	9.6 ± 0.9*^†^	0.353	< 0.001
LV mass index, g/m^2.7^	49.2 ± 10.6	45.9 ± 10.9*	40.8 ± 11.9*^†^	0.350	< 0.001
LV ejection fraction, %	63.9 ± 4.1	63.6 ± 5.3	63.9 ± 5.7	− 0.033	0.581
E/A < 1, n (%)	74 (64.9)	74 (64.3)	31 (60.8)	0.000	0.999
HT, n (%)	65 (57.0)	46 (40.0)*	13 (25.5)*	0.265	< 0.001
SBP, mmHg	140.4 ± 19.9	137.6 ± 19.2	129.6 ± 19.1*^†^	0.180	0.002
DBP, mmHg	79.8 ± 12.6	75.1 ± 10.8	74.2 ± 10.3	0.073	0.226
Type 2 DM, n (%)	28 (24.6)	16 (13.9)*	0 (0.0)*^†^	0.249	< 0.001
FBG, mmol/L	6.2 ± 1.9	5.8 ± 1.7	5.1 ± 0.9	0.161	0.054
TC, mmol/L	4.6 ± 0.8	4.0 ± 0.9*	3.8 ± 0.8*	0.206	0.006
LDL-C, mmol/L	2.7 ± 0.7	2.3 ± 0.7	2.3 ± 0.7	0.059	0.438
HDL-C, mmol/L	1.3 ± 0.5	1.4 ± 0.4	1.7 ± 0.5*^†^	− 0.301	< 0.001
TG, mmol/L	1.1 ± 0.5	0.9 ± 0.3	0.9 ± 0.4	0.115	0.053
Gout, n (%)	0 (0.0)	2 (1.7)	0 (0.0)	0.007	0.911
eGFR, ml/min/1.73m^2^	79.7 ± 16.0	81.0 ± 16.1	88.6 ± 15.4*^†^	− 0.190	0.001
CHD, n (%)	24 (21.1)	24 (20.9)	7 (13.7)	0.074	0.216
Stroke, n (%)	12 (10.5)	19 (16.5)	5 (9.8)	0.008	0.892
LABA, n (%)	85 (74.6)	93 (80.9)	42 (82.4)	− 0.112	0.062
LAMA, n (%)	59 (51.8)	71 (61.7)	37 (72.6)	− 0.093	0.120

Values presented as mean ± SD or n (%).*p < 0.05 versus Overweight obesity group; †*p* < 0.05 versus Normal weight group. COPD, chronic obstructive pulmonary disease; BMI, body mass index; FEV1, forced expiratory volume in one second; FVC, forced vital capacity; RV, residual volume; TLC, total lung capacity; DLCO, diffusing capacity of carbon monoxide; RV, right ventricular; RA, right atrial; LA, left atrial; LV ESD, left ventricular end-systolic diameter; LV EDD, left ventricular end-diastolic diameter; IV, interventricular; LV PWT, left ventricular posterior wall thickness; LV, left ventricular; E/A: transmitral peak early (E) and late (A) filling velocity ratio; HT, hypertension; SBP, systolic blood pressure; DBP, diastolic blood pressure; DM, diabetes mellitus; FPG, fast plasma glucose; TC, total cholesterol; LDL-C, low density lipoprotein cholesterol; HDL-C, high density lipoprotein cholesterol; TG, triglyceride; eGFR, estimated glomerular filtration rate; CHD, coronary heart disease; LABA, Long-acting β2 adrenergic receptor agonist; LAMA, Long-acting muscarinic antagonist.

### Underweight, echocardiographic, lung functional and clinical parameters in patients with severe COPD

Correlation analysis for the underweight with echocardiographic parameters was underwent in COPD patients with severe lung functional reduction (n = 280), as considering that the changes in echocardiographic parameters and reduced BMI existed in COPD patients with severe lung functional reduction. Univariate correlation analysis showed that underweight was related to reduced LA diameter, LV mass index and wall thickness, impaired lung function, decreased plasma total cholesterol, systolic blood pressure, and prevalence of hypertension and type 2 DM in COPD patients with severe lung functional reduction; Meanwhile underweight was related to increased RV and RA diameter, HDL-C and eGFR in COPD patients with severe lung functional reduction (All *P* < 0.05, Table 2).

Dichotomized variate regression analysis showed that underweight was independently associated with reduced LV mass and wall thickness, dilated RV and decreased lung function in COPD patients with severe lung functional reduction (All *P* < 0.05, Table 3).

**Table 3:** Regression analysis of parameters associated with underweight (BMI < 18.5 kg/m^2^) in patients with severe COPD (n = 280).

Variable	β	SE	Wald	*P* value	95% confidence
FEV1% pred	− 0.346	0.173	4.010	0.045	0.504–0.993
FEV1/FVC	− 0.059	0.097	0.373	0.541	0.779–1.140
RV/TLC	0.046	0.138	0.110	0.740	0.799–1.370
DLCO% pred	− 0.055	0.869	0.004	0.949	0.172–5.200
RV diameter	0.120	0.507	4.002	0.048	0.328–2.398
RA diameter	0.267	0.261	1.050	0.305	0.784–2.177
LA diameter	− 0.085	0.410	0.043	0.836	0.487–2.434
IV septum	− 2.469	1.149	4.617	0.032	0.009–0.805
LV PWT	− 4.056	1.833	4.895	0.027	1.589–2.099
LV mass index	− 0.447	0.208	4.593	0.032	0.425–0.963
HT	− 2.993	2.214	1.827	0.176	0.001–3.845
SBP	− 0.061	0.055	1.239	0.266	0.844–1.048
Type 2 DM	− 20.973	6249.125	0.000	0.997	0.001–1.594
TC	− 0.556	1.024	0.295	0.587	0.234–1.298
HDL-C	3.426	2.245	2.330	0.127	0.378–2.505
eGFR	0.185	0.101	3.332	0.068	0.986–1.468

COPD, chronic obstructive pulmonary disease; BMI, body mass index; FEV1, forced expiratory volume in one second; FVC, forced vital capacity; RV, residual volume; TLC, total lung capacity; DLCO, diffusing capacity of carbon monoxide; RV, right ventricular; RA, right atrial; LA, left atrial; IV, interventricular; LV PWT, left ventricular posterior wall thickness; LV, left ventricular; HT, hypertension; SBP, systolic blood pressure; DM, diabetes mellitus; TC, total cholesterol; HDL-C, high density lipoprotein cholesterol; eGFR, estimated glomerular filtration rate.

## Discussion

The present study shows that lowered BMI, LA diameter and LV mass index, and increased RV diameter existed in COPD patients with severe lung functional reduction. The results indicated that lower BMI was independently associated not only with increased RV diameter and impaired lung function, but also it is related to a decreased LV mass in elderly Asian population with severe COPD.

The present study confirmed that the elderly COPD patient population with its severe forms is more common among males than females. Chronic inflammation leads to fibrosis of lung parenchyma in the pathological process of COPD. The elasticity of respiratory bronchioles gradually decreases. The air in alveoli can not be completely discharged during the expiratory period. It gradually leads to the increase of residual air in the alveoli, formation of emphysema and dysfunction of airway ventilation. In the late stage of COPD, alveolar tissue fibrosis and capillary destruction in the lung further lead to declined pulmonary diffusional function. The destruction of pulmonary vascular bed gradually leads to increase of lung artery resistance, pulmonary hypertension and right-sided cardiac atrial and ventricular dilation and dysfunction, and exhibiting liver and gastrointestinal blood congestion, indigestion, absorption dysfunction, malnutrition and decreased BMI^22^. Our present results showed that underweight and the right-sided cardiac ventricular dilation were observed commonly in severe COPD. This finding is consistent with previous studies^4,23,24^. However, our present study replenished that underweight was independently related to the right-sided cardiac ventricular dilation and impaired pulmonary function in this group of COPD patients with severe lung functional reduction.

With progression of COPD and pulmonary functional decline, the subsequent hypoxia activates sympathetic nervous and renin-angiotensin-aldosterone systems. The respiratory muscles are forced to do more work for maintaining adequate lung ventilation, which increases more energy consumption and produces more inflammatory molecules^25^. It raises the catabolism of glucose, fat and even protein, consequently leading to sarcopenia of skeletal muscles and increased systemic inflammation with resultant bodyweight loss, lower BMI^26,27^. It may also cause sarcopenia of cardiac muscles with decreased LV mass. At the same time, due to the destruction of pulmonary vessels, the blood volume from right-sided to left-sided heart (the left-sided cardiac preload) is reduced^11,12^. Then, the decreased left-sided cardiac ventricular mass and thickness would result, and the blood supply for peripheral tissues will further decline, which is another important reason for the decrease of BMI. On the basis of our knowledge, this is the first time that our present study confirmed that pulmonary functional impairment and reduced LV mass is associated with lower BMI in the elderly COPD with severe lung dysfunction. This study suggests that echocardiographic parameters can be considered to add into future revised COPD prognostic assessment scales^28,29^. Low BMI associated cardiac remodeling may provide a useful marker for prognosis of COPD patients. However, our results from Asian population are not consistent with a previous study, which reported that LV hypertrophy existed in COPD^30^. The previous study was consisted of relatively younger patients mainly with mild moderate COPD^30^. The underlying causes for these differences are still unclear. However, the discrepancies may come from the different studied populations and measurement methods for the LV mass. In our study, the decreased LV mass and thickness were found usually in the elderly COPD with severe lung dysfunction.

Although previous studies reported that low BMI can lead to lung dysfunction, and is closely related to the occurrence and development of COPD^31–33^, other investigations confirmed that low BMI is not a causal factor for the development of COPD^34,35^. In contrary, it suggested that lower BMI and its associated cardiac remodeling may be considered to result from the consequences of increased expenditure of energy, reduced nutritional intake and increased inflammation due to COPD and its complicated pulmonary heart disease^34,35^. Therefore, we advise that moderately active treatment of COPD may improve the patient’s lung function and prognosis, and prevent progression of the disease into advanced stage with complicating lower BMI and cardiac adverse structural remodeling. Treatments including appropriate nutritional supplementation may help to improve the prognosis of COPD patients. On the other hand, it showed that COPD patients with higher BMI have better lung function and prognosis. It seems that overweight and obesity may partly even protect lung function and prevent the development of COPD, which is called “obesity paradox”^36,37^. Nevertheless, this inverse relationship between body mass and pulmonary poor prognosis in COPD could be explained by a speculation that a better lung function with its subsequent less energy exhaustion, anorexia and inflammation may contribute to the bigger body mass due to the relatively moderate severity of the lung diseases^36,37^. On the contrast, previous studies have demonstrated that morbid obesity can increase risk of asthma and obstructive sleep apnea or hypopnea^38,39^. Our present study also found that the COPD patients with relatively increased BMI have a better lung function, nevertheless, they are with more metabolic cardiovascular disorders and left-sided cardiac adverse hypertrophic remodeling. In addition, the prevalence of E/A reversal was not found to be significantly different among BMI categories in severe COPD patients. The E/A reversal assessment alone may not precisely reflect cardiac LV diastolic dysfunction.

The present study has some limitations. It was a cross-sectional investigation. Data were collected retrospectively. The effects of unavoidable confounding factors need to be considered. In this study, the health assessment scale was not used to estimate the nutritional status in the subjects. It is still unclear whether malnutrition involves the association of low body weight with the cardiac remodeling in the COPD patients. Therefore, the observed findings in this study would be needed to reconfirm by future prospective studies. This study was conducted in elderly patients with COPD. The results should be carefully extrapolated to other populations.

## Conclusion

The present study found that low BMI, decreased LV mass and LA diameter, and increased RV diameter existed more often in elderly COPD patients with severe lung dysfunction. Low BMI was not only independently related to RV dilation and lung functional impairment, but also it was associated with decreased LV mass in elderly Asian population with severe COPD.
